# Retraction: Multi-walled carbon nanotubes decorated with palladium nanoparticles as a novel platform for electrocatalytic sensing applications

**DOI:** 10.1039/d5ra90051d

**Published:** 2025-05-02

**Authors:** Mehdi Baghayeri, Hojat Veisi, Hamed Veisi, Behrooz Maleki, Hassan Karimi-Maleh, Hadi Beitollahi

**Affiliations:** a Department of Chemistry, Faculty of Science, Hakim Sabzevari University P. O. Box 397 Sabzevar Iran m.baghayeri@hsu.ac.ir +98 5714003170 +98 5714003325; b Department of Chemistry, Payame Noor University 19395-4697 Tehran Iran; c Student Research Committee, Shiraz University of Medical Sciences Shiraz Iran; d Department of Chemistry, Graduate University of Advanced Technology Kerman Iran; e Environment Department, Institute of Science and High Technology and Environmental Sciences, Graduate University of Advanced Technology Kerman Iran

## Abstract

Retraction of ‘Multi-walled carbon nanotubes decorated with palladium nanoparticles as a novel platform for electrocatalytic sensing applications’ by Mehdi Baghayeri *et al.*, *RSC Adv.*, 2014, **4**, 49595–49604, https://doi.org/10.1039/C4RA08536A.

The Royal Society of Chemistry hereby wholly retracts this *RSC Advances* article due to concerns with the reliability of the data.

In the XRD data in Fig. 2, there are sections with repeating patterns. In the FT-IR spectra in Fig. 3, there are duplicating segments in traces c, d and e. In Fig. 4, there are duplicating sections between trace a and c, and between trace b and c.

The authors were contacted but did not provide a response to the concerns.

Given the significance of these concerns, the Editor has lost confidence that the findings presented in this paper are reliable.

This retraction supersedes the information provided in the Expression of Concern related to this article.

The authors were informed about the retraction of the article. Hojat Veisi and Behrooz Maleki have not agreed with the decision, the other authors have not responded.

Signed: Laura Fisher, Executive Editor, *RSC Advances*

Date: 25th April 2025

